# Ethical Design in the Internet of Things

**DOI:** 10.1007/s11948-016-9754-5

**Published:** 2016-01-21

**Authors:** Gianmarco Baldini, Maarten Botterman, Ricardo Neisse, Mariachiara Tallacchini

**Affiliations:** 1 European Commission, Joint Research Centre, Ispra, Italy; 2 GNKS Consult, Rotterdam, The Netherlands; 3 Universita’ Cattolica S.C., Milan, Italy

**Keywords:** Personalization, Privacy, Ethics, Agency, Users’ empowerment

## Abstract

Even though public awareness about privacy risks in the Internet is increasing, in the evolution of the Internet to the Internet of Things (IoT) these risks are likely to become more relevant due to the large amount of data collected and processed by the “Things”. The business drivers for exploring ways to monetize such data are one of the challenges identified in this paper for the protection of Privacy in the IoT. Beyond the protection of privacy, this paper highlights the need for new approaches, which grant a more active role to the users of the IoT and which address other potential issues such as the Digital Divide or safety risks. A key facet in ethical design is the transparency of the technology and services in how that technology handles data, as well as providing choice for the user. This paper presents a new approach for users’ interaction with the IoT, which is based on the concept of Ethical Design implemented through a policy-based framework. In the proposed framework, users are provided with wider controls over personal data or the IoT services by selecting specific sets of policies, which can be tailored according to users’ capabilities and to the contexts where they operate. The potential deployment of the framework in a typical IoT context is described with the identification of the main stakeholders and the processes that should be put in place.

## Introduction

Public awareness of privacy risks in the Internet has increased while governing bodies are working on new sets of regulations to address concerns and gaps in the digital domain.

In Europe, the EU has proposed a new Data Protection Regulation (DPR) (European Commission 2012), which has the goal to address some of the most pressing data protection issues created by new technologies (e.g., smart phones or cloud computing). Particularly it aims at ensuring that the personal data of individuals are protected no matter where or what form of processing is undertaken, defining “personal data” as any data that can be related to individuals, which means that the definition can extend to large parts of the IoT.

In the USA, various initiatives have addressed the need for users’ privacy including the 2012 report (White House 2012), which had the objective to define a framework for protecting privacy of the consumer in a networking world. One of the key components of the report is the Consumer Privacy Bill of Rights, which is based on the definition of Fair Information Practice Principles (FIPP), which include (a) *respect for context* where consumers have a right to expect that companies will collect, use, and disclose personal data in ways that are consistent with the context in which consumers provide the data and (b) *Individual Control* where Consumers have a right to exercise control over what personal data companies collect from them and how they use it. There are two key principles which will be addressed in the framework proposed in this paper. See also the recent paper from Weber (2015) for a study of the main challenges to guarantee data protection laws and the concept of privacy in an increasingly networked world.

Most of these initiatives originated by privacy risks in the Internet world and in relation to “big data”, but we claim that in the evolution of Internet to the Internet of Things (IoT), these risks are likely to become even more relevant, due to the large amount of data collected and processed by the “Things”. Such data may be related to persons, their daily activities and the increased relationships between the “digital” and “real” worlds due to new IoT devices like wearable sensors. There have been many definitions of the IoT. In this paper, we present two of them. In Guillemin (2009) a definition of the IoT is given as follows: “The Internet of Things allows people and things to be connected Anytime, Anyplace, with Anything and Anyone, ideally using Any path/network and Any service”. In Haller et al. (2009), the Internet of Things is defined as “a world where physical objects are seamlessly integrated into the information network, and where the physical objects can become active participants in business processes. Services are available to interact with these ‘smart objects’ over the Internet, query their state and any information associated with them, taking into account security and privacy issues.” Both definitions point to the pervasiveness of the IoT and to an improved integration between the digital and the real world. While we acknowledge that IoT can be considered an evolution of the Internet and the boundaries between the two worlds can be blurred, we include in the IoT new categories of electronic devices, which did not exist in the conventional Internet where users access the web though a personal computer interface. These categories include (a) new small electronic devices which can be worn by a person (e.g., wearable sensors); (b) smart cars which are connected among themselves and a fixed infrastructure to support intelligent traffic and safety applications (e.g., collision avoidance); (c) machine to machine systems which are used in an industrial context but also to make the home more intelligent and responsive to human beings (e.g., the concept of the smart home); (d) remote healthcare systems which can monitor the health of an individual at any time. The list could continue with new devices and applications which are not even foreseeable now. The main features shared by these different categories of devices are the almost continuous connectivity through a wide range of wireless communications standards (e.g., WiFi, UMTS, LTE, ZigBee) and the capacity to collect data from the real world (e.g., camera) or to act on the real world (e.g., actuators like a domotic system to regulate the temperature of the house), including from an individual (e.g., a sensor collecting blood pressure readings at any time) or data that often can be related to each other through identification of time and (geo)location. Another component of the IoT is the connection of the IoT device to the Cloud, where the data can be collected or aggregated, and where sophisticated analytical algorithms can be applied to them to identify users’ behavioral patterns (which can also become a privacy risk). See (Weinberg et al. 2015; Article 29 Working Party 2014) for a more detailed analysis of the distinguishing characteristics of the Internet of Things.

Several claims have been made that the concept of privacy should be revisited, as the amount of collected data from the IoT will be too difficult to control—and the complexity becomes even higher when attempting to determine which data are personal and which are not. Some tensions exist between the technical measures for individual privacy enhancement and the business opportunities for digital-related commercial activities that correlate different sets of data collected from the IoT devices, and then explore ways to monetize them. This opportunity to monetize data using data analytics is one of the reasons why the market success of Privacy Enhancing Technologies (PET) and the application of Privacy Enhancing Measures (PEM) remain limited (Cave et al. 2011). In other words, the reasons for the business failure of PET and PEM in today’s market, or for privacy protection solutions in general, may not only be related to technical reasons or lack of knowledge, but also (and maybe even more) to misplaced incentives from an economic point of view (Alessandro 2004).

For example, see Cas (2005) for an analysis of privacy tradeoffs in pervasive computing. While large amounts of data was difficult to relate to individuals in the past, data collected from the physical world has currently become more and more relatable, and both regulators and the public pay increased attention to the protection of privacy and private data. In particular, public concern and awareness of privacy implications are growing as to the use of big data analytics on data collected through the Internet and the IoT (Anton et al. 2010). For example, the new European privacy legislation, that is about to become adopted, *inter alia* includes requirements for facilitating easy access by persons to personal data collected about them, and opt-in, rather than opt-out and silent, consent for electronic data collection. Whereas it is already difficult today, it will be even more difficult in the future to determine what data relate “enough” to persons to be considered “personal,” and thus within the scope of the new legislation, as it is clear that much more data can be related to persons than in the past. As the amount of data relatable to individuals is increasing and what should count as personal data is controversial, interested individuals should be able to decide how to frame the boundaries of their private information domain.

An important aspect highlighted by Nissenbaum (2015) is that privacy protection should be related to the “context” where the user operates. Contexts are shaped by technology, business practice, and industry sector or other features like geographic location, relationship, place, space, agreement, culture and religion and existing regulatory frameworks. While this is important for the Internet, it is even more relevant for the IoT, where contexts can dynamically change (e.g., office or home environment). We believe that support for context should be an essential feature of any approach to address privacy aspects or in general the relationship between users and the IoT. In fact, this is a central feature of the “Ethical design” concept and the related framework presented in this paper.

In addition, the Internet and the IoT user’s individual decision process with respect to privacy is affected and hampered by multiple factors, as described in Acquisti and Grossklags (2005): (a) incomplete information on the consequences of an action (e.g., search on a person or introduction of data) affects a privacy decision; (b) the complete set of needed information to make a rational choice could be so large that the user may not be able to access the IoT service in an effective way; (c) even in the case of complete information, psychological biases may force users to make wrong decisions about privacy.

To summarize, we believe that the following legal-socio-economic aspects and related challenges will be relevant in the evolution of the IoT regarding users’ privacy protection: (a) there is a trade-off between the market needs for data collection and correlation to support innovation and the business success of the IoT systems and applications (for both the public and private sectors) and the protection of users’ data; (b) the cost of certifying and implementing PET, or other solutions to ensure proper care in collection, storage and retrieval of data; (c) the accountability of the IoT applications regarding users’ privacy and (d) support for the context where the user operates. The challenges described above are further discussed in “Challenges and Processes for Ethical Design in the IoT” section.

Beyond privacy, other aspects will become more relevant in the future in the interaction between the user and the IoT. One important aspect is the Digital Divide. Persons have different capabilities when interacting with Internet or, in the future, with the IoT. Children can be more vulnerable to malicious users when navigating the web. Elderly people may have more difficulty to adjust to new technologies like Smart Cars. As described in Weber (2013), a right of access to the IoT infrastructure must be granted, which is equitable and non-discriminatory by all interested stakeholders (e.g., citizen, businesses). Access to infrastructure encompasses open access to the system, open standards, open-source software and wide-spread availability of access points to support affordability of access and its communication possibilities.

Another aspect is the control on the flow of data coming from the IoT. When new collaborative applications will be deployed in the IoT, the users of the applications may have a limited control on the flow of data or requests (e.g., to participate or register to a specific service) coming to them (Gottschalk-Mazouz 2013). Again, the problem of different users’s capabilities to manage these requests is another important challenge in the IoT. Already web advertising often sends unsolicited requests to users. How can the users control such flow of data or requests when more sophisticated applications will be deployed in the IoT?

Whereas the future is unpredictable by definition, it is clear that increased data generation by the emergence and further immersion of the IoT, in combination with innovations and advancement of effective big data analytics, will change the landscape in which people position themselves with respect to the economics shaping the evolution of the Internet toward the IoT even further over the coming years. To address this issue, and while acknowledging that a legal framework alone will not be able to ensure this balance, we propose in this paper the expression “Ethical Design” for future IoT devices and services, where “ethical choices” made available to users within the digital architecture become an added value that users are willing to pay for, and that will ensure the needed balance. Similarly to other areas of the “ethical market”—such as ethical funds, cruelty free foods and cosmetics, and fair trade items—“ethically oriented” (or ethically friendly), ICT may be seen as a new desirable feature for the digital life (Arendt 1958). In this paper, we focus on the market sustainability of solutions to respect the rights of the citizen (e.g., privacy rights) in the use of the IoT and we do not address “ethics” aspects in a wider context. In other words, we are not going to address broad ethical themes at the roots of technological development, concerning freedom, justice, identity, values and ultimately contested world views; instead, we look at how the IoT can be designed to allow users/citizens to choose their own values as a matter of moral entitlement – well beyond the current users’ preferences policies. For instance, some authors are already arguing that access to raw data should be considered as a moral right (Lunshof et al. 2014).

We discuss how this market could be sustained and what role for regulation can be envisaged. We will describe how Ethical Design can mitigate the challenges described above. Then, the paper presents a practical implementation of the proposed Ethical design model through a policy-based framework where the main stakeholders are identified.

Note that in this paper, we use the term “ethical design” from an engineering point of view to highlight that the IoT engineers promote a design of the IoT, which is respectful of the rights of the citizens instead of being only driven by economic considerations. Similar terms like “defensive or anticipatory design” can also be used.

The structure of the paper is the following: Section “A Model for Ethical Design in the IoT ” describes the concept of the “Ethical Design” in the IoT, identifies the potential challenges to be overcome and the main processes to support the development and deployment of an “Ethical Design” in the IoT. Section “Policy-Based Approach for Ethical Design ” describes the proposed technical implementation of the “Ethical Design” through a policy-based framework. Finally, “Conclusions and Future Developments” section offers some concluding reflections.

## A Model for Ethical Design in the IoT

### Ethical Design and Human Agency

In this paper, the concept of “Ethical Design” is introduced to refer to the IoT products (e.g., devices and applications) which are designed and deployed to empower users in controlling and protecting their personal data and any other information. This means that users should be enabled to establish and freely shape their value-laden choices while interacting within the IoT. While these choices are normally embedded in the algorithms as a result of the decision of programmers and developers, an “Ethical design” would make these decisions directly available to the user.

These choices certainly include different degrees and aspects of privacy and data protection, but can extend to numerous other ethical choices that can emerge through newly established relations amongst connected Things. The similarity is, therefore, with other “ethical products” that motivated and socially responsible consumers may be willing to buy in the marketplace. Indeed, ethical business has managed to create sustainable business models embedded in ethical values. As an “ethical” IoT device or service is designed to ensure a higher level of individual freedom and choice, the additional cost for the implementation and deployment of the “ethical” framing is justified and is likely to appeal to interested users, with the creation of a specific market.

In the proposed model, the design is seen as “ethical” because it is built to empower and entitle users to frame their ethical choices whenever value-laden issues emerge. To this end, values are neither imposed nor pre-identified, but remain open to individuals’ customization of ethical options. In other terms, values are proposed and framed as an individual right of choice in the ICT architecture. Fostering and enhancing the individual ability to freely determine the values that will orient and bind interactions in the IoT is also a response to a major challenge posed by the IoT, namely its tendency to reduce human agency, awareness and reflexivity as a consequence of the deterministic deployment of Things. Indeed, agency (Arendt 1958) refers to the human capacity to intentionally reflect and act as a self-governed subject rather than as an automated mechanism in the interactions with the IoT. These human features imply use of intentionality and awareness, freedom, control on thoughts and acts, as well as their human limits.

As these human characteristics are at risk of being unlearned if not properly cultivated and practiced, devices and products fostering these dimensions can be considered as performing an ethical function; also, there is a market for “ethical” IoT products which not only protects the privacy of the individual, but supports human agency as well. In Trappeniers et al. (2009) a similar form of empowerment for end-users with varying degrees of programming skills in their interaction with the IoT is presented. In the cited work, the focus is on enabling ordinary people to easily create, setup and control applications in their smart living environments as well as in the public IoT space on the basis of available IoT services and devices. Obviously, the collaborative development of such application requires a mutual trust among the participants. Trust is boosted by a recognition of personal needs; by transparency in how things are organized—namely in a way that clearly shows that relevant measures have been taken to meet those needs—; and by accountability in ensuring that the responsibilities are clear (Kounelis et al. 2014).

The concept of Ethical design in the IoT shares some similarities with the concept of “anticipatory ethics” expressed in Brey (2012). Indeed, we agree that this concept should become a component of the design of emerging technologies and lead to better ethical outcomes and socially responsible technologies. A similar example to the concept of Ethical Design presented in this paper is the Named Data Networking project presented recently in Shilton (2015). This project seeks to understand how anticipatory ethics might support social values and embed them in the design in a new Internet architecture. Even if the type of technology is slightly different because it focuses on new protocols for Internet architecture and our proposal is based on a policy management framework, we believe that the underlying principles of anticipatory ethics are quite similar. In fact, both approaches aim at openly and publicly discussing what are the agreed values before they become embedded and encrypted in digital architectures. The need for an ethical approach in the design of the IoT to mitigate the risk of security vulnerabilities has also been highlighted by Cavelty (2014) and we believe that our Ethical design concept goes in the same direction.

In their Opinion on the ethics of security and surveillance technologies (EGE 2014, 32), the European Group for Ethics in Science and New Technologies to the European Commission has made use of the concept Privacy in Design (as distinct from Privacy by Design) as the process of “raising awareness about the processes through which values and norms become embedded in technological architecture. Privacy in design looks at the normativity of structural choices in an effort to promote transparency and protect rights and values of the citizens”.

Indeed, while in the by-design approach, users are provided with predefined forms of protection, in the in-design approach digital architectures are opened up to users—which also implies a shift in understanding privacy as a “right” rather than a paternalistic legal protection (Pereira and Tallacchini 2014).

In summary, the IoT products based on an Ethical design should be based or have the following features: (a) capability to provide control of the collection and distribution of data or services related to the user. In other words, they aim to support the ethical capabilities of human beings such as agency, awareness and reflexivity (requiring transparency on how data are collected and distributed); (b) capability to enforce different regulations or cultures along the dimensions of time (e.g., cultural or regulatory changes) or space (e.g., different nations); (c) able to supporting dynamic contexts (e.g., house, office) and (d) able to perceive, identify and support relationships, which require ethical choices.

The concept of Ethical Design could be implemented using different technologies. In “Policy-Based Approach for Ethical Design” section, we describe a potential implementation of the Ethical Design concept using a policy-based framework.

### Challenges and Processes for Ethical Design in the IoT

In this Section, we present the socio-economic benefits of Ethical Design in the IoT and how our proposal can address or mitigate the challenges introduced in “Introduction” section and summarized in Table 1. In the rest of this paper, we describe how each of the challenges described in Table 1 are addressed.

**Table 1:** Internet of things challenges

	Challenge	Description
1	Economic incentives for data protection of the user are not directed to the user	Economic incentives for data protection of the user are limited to the businesses creating the IoT applications and devices
2	Incomplete information on the consequence of data disclosure	The user has often incomplete information about the consequences of disclosing data either voluntarily (e.g., providing data) or involuntarily (e.g., collection of position information). This lack of information affects each privacy decision. The incomplete information can also be a consequence of a limited perception by the user (e.g., the digital divide problem). In the IoT, this issue could be more relevant than in the Internet as the physical world information (e.g., physical position) could increase the information space. This is related to a concept of *transparency* on how disclosed data is used by the developer of the IoT system or application
3	Too large information space about the consequence of data disclosure	The complete set of needed information to make a rational choice could be so large that the user may not be able to access the IoT service in an effective way
4	Psychological biases	For example, the perception of immediate benefits (e.g., free access to an IoT service or application) can impact the long-term negative impact (e.g., risk to users’ privacy)
5	Trade-offs between businesses needs to collect and process data and rights to privacy	There is something of a tension between the market’s needs for data collection and correlation to support innovation and the business success of the IoT systems and applications (for both the public and private sector) and the protection of users’ data. While government (e.g., regulators’ bodies) may support the balance on one or another direction, one significant challenge is to design and apply regulations in a very dynamic environment where the life-cycle of the IoT applications in the market can be much shorter than the regulatory process
6	Cost of implementing privacy enhancing or data protection solutions	The costs of implementing PET, or other solutions to ensure proper care in collection, storage and retrieval of data. Who is going to support these costs? For example: that the willingness of the user to pay for the service, or the political will to ensure societal guarantees enforced through legislation
7	Accountability	The accountability of the IoT applications regarding users’ privacy. Who is going to be legally accountable for the user’s data? As seen in recent events, a data breach can be extremely damaging to a business company from an economic point of view. Are PET producers responsible for privacy breaches or the application where the PET is applied? Or the users themselves?
8	On-line and off-line identity	It is difficult to separate the on-line information from the off-line information and their linkage can generate privacy breaches
9	Digital divide	Users have different set of capabilities in accessing the IoT devices and applications. Depending on their level of technical proficiency, users have different levels of perceptions of the privacy risks or different understanding of the requests sent to them through the IoT
10	Conformance to regulatory frameworks	The definition, implementation and conformance to regulations in this context can be hampered by two factors: (1) the speed of the evolution of the IoT can be faster than the regulatory process itself, so that regulations can be moderately effective when they are enforced, (2) already deployed IoT systems and devices may require significant rework or replacement (e.g., recall of the IoT devices) which can be very expensive for companies
11	Support for dynamic context	The use of the IoT services and devices and the processing and storage of personal data may change depending on the context as recommended in Nissenbaum (2015)

The first challenges we want to address in this paper are (1) and (5) from Table 1. Poor application of security (or privacy) measures may not depend on technical reasons or lack of knowledge, but rather, from an economic point of view, be the outcome of conscious choices resulting from misplaced incentives. In the case of privacy, for manufacturers and developers the main economic incentives depend on creating applications or devices where (especially) users’ data can be collected, rather than protected. The proposal for Ethical Design specifically addresses these challenges by developing and promoting products with both embedded protective technical solutions (as described in Policy-Based Approach for Ethical Design  section) (for unskilled users) and advanced options of control for more skilled users. For the user who is aware of both short term and long term benefits, the added value of this Ethical design justifies its higher costs (due to implementation of the technical solutions), and therefore its adoption and purchase [see Table 1, challenge (6)]. It has to be highlighted that this does not contradict the long-term strategy of many companies involved in the Internet and the IoT markets. As Eric Schmidt (CEO of Google) argued in Schmidt (2010), businesses should be focused on users and their needs, which is the same objective of the Ethical Design concept. Through an Ethical Design of the IoT products and services, from the outset developers and manufacturers think ahead of the curve towards what a responsible strategy should be—and, thus, will ensure viability of these products and services in a social environment, and relevance for the years to come. Because of the potential societal benefits, the government could be involved in the adoption of the Ethical Design concept through regulations or through standardization activities where key elements of Ethical Design could be defined.

A potential issue is that these incentives are not broadly understood and not always recognized both by private and public stakeholders. Altogether, it is clear that these incentives need to be identified and presented in such a way that developers as well as marketers of products and services can translate them into features, requirements and terms that are well understood by their customers.

The following set of processes expresses the drivers and challenges at several levels for the design and deployment of the IoT products based on Ethical Design (i.e., approach/process/product):

1. *Understanding the need for and value of trust* in the global, networked, knowledge-based society at the level of public and private decision-makers, thus ensuring preparedness to invest in this at the highest levels. This means that the design of the IoT should include components to support trust in the use of the IoT services and mutual trust among the IoT users. For example, the provision of authentication functions to ensure that only authenticated and certified entities can provide the IoT services is an element to build trust because the user can have confidence in using those IoT services and authenticated users can be held accountable and liable. Here the role of public (e.g., government) or private decision makers (e.g., industrial forum) is essential because these decision makers can enforce regulations or design solutions (e.g., embedded in standards) in the market. Another example is explicitly respecting data minimizations, data collection relating to purpose, and transparency of data collection and distribution in the first place, thus limiting “surprises” in terms of use of personal data.
2. *Translating these needs and values into an Ethical Design of the IoT products and services*. In order to “create” an Ethical Design, the involvement of the user in the design phase will go beyond usability: it is about “user experience design” in the widest sense, as it also entails experiences such as trust and comfort, including awareness of relevant value-laden choices. As users are asked to share their experiences in an environment that is to roll out towards the future, with an impact that will vary depending on the level of immersion in our environment, it is important that users are informed and invited to think ahead, by engaging them in the design phase.
3. *Demonstrating that these needs and values are taken into account*, in the context of specific trade-offs between ensuring needs and values (e.g., trust) on the one hand, and ease and quality of experience and affordability on the other hand. One thing is that manufacturers design “connected things” and services using the IoT in such a way that it responds to the needs and values of users. Indeed, certain ethical choices, as it happens with other “ethical products”, are made by individuals not only as consumers, but also as citizens: namely as bearers of broader civic values that they want to protect in society. One step further is that they act in a way that can be grasped and shared by users. This goal can be only achieved through a combination of (a two-way) education (“what should matter to whom, and why”), and simplicity and transparency in design, so that people can comprehend what “connected things” do and what happens with the information collected, in terms of “what is collected on me” (what data are registered, anyway), “how it is stored” (how easy would it be to correlate specific data with me) and “how it can be accessed” (is it properly protected against abuse, is it noted when it is accessed, etc.). These considerations specifically address the challenges (2), (3) and (9) in Table 1. The definition of open standards is one of the ways that can enhance trust through transparency. The creation of processes for the certification of the IoT products with Ethical Design is another essential step to be executed in collaboration with standardization bodies, manufacturers, certification centers and service providers.
4. *Establishment of a clear framework for transparency and accountability*. While the existing legal framework to protect citizens and consumers was not developed to include the IoT, we need to establish an IoT environment that reflects respect of the existing legal framework, while pre-empting the evolution of the regulatory framework to reflect a changing world (challenge (10) in Table 1). In this context, connected IoT devices based on Ethical Design with commonly agreed standards for trust and privacy allowing the IoT to innovate and grow also in a beneficial way for society are an important element to support this framework. In addition, this step is essential to address challenge (7) on Accountability in Table 1.

In summary, Ethical Design for developers and entrepreneurs may bring a number of advantages: (a) it reduces business risks for investments in products and services from a legal perspective; (b) it supports businesses in a long term relationship with consumers who want to buy ethically-framed products and services and are willing to use those that better meet their needs; (c) it helps create a society where people feel good about using the IoT and have a relatively high level of trust towards it, thus keeping “transaction costs” overall at a relatively low level. Even more so: as the “future capital” will be largely based on usable data, this concept—how to benefit in a legal and ethical way from data generated through the IoT products and services—will lead to even more solid data access with no (or little) drive for people wishing to “opt out”.

Once these needs and challenges are identified, the next step is to design and deploy the technological framework, which implements the Ethical Design concept. This is the objective of the framework described in the next section.

## Policy-Based Approach for Ethical Design

### Main Concepts

In this section, we describe a potential implementation of the Ethical Design framework described in the previous sections through a policy-based approach. By policy here we refer to the normative framework, namely the set of principles and rules, adopted and implemented within a digital architecture or device, and regulating their behaviors in their relations with users, their expectations and rights.

The choice and definition of the technical framework to support the concept of Ethical Design is based on the challenges described in “Challenges and Processes for Ethical Design in the IoT” section, which are translated into the following technical features: a capability to define rules for the access to data and the IoT services both in a manual and an automatic way, support for a digital divide, capability to embed the rules in the IoT devices with minimal performance impact and support for dynamic change of the context. Among the various technical approaches available in literature, these features point out to a policy management framework. Other solutions are also possible. For specific privacy aspects, see Heurix et al. (2015) for a taxonomy of privacy enhancing technologies. How the framework proposed in this paper compares to other frameworks or design solutions is described in “Analysis and Comparison with Other Frameworks” section.

We propose to implement the Ethical Design concept using the Model-based Security Toolkit (SecKit), which is a sophisticated policy-based framework already introduced in Neisse et al. (2015) and Neisse et al. (2014a). The SecKit has the capability of empowering users in the usage of the IoT services and resources, including their personal data. The usage is regulated through *profiles*, which consist of sets of security policy configuration rules that specify the conditions when a set of *enforcement* policy templates should be activated. For example, a profile can be specified to restrict the amount of user information accessible to the IoT devices (*enforcement*) when the user is in a public space that is considered to be a potentially unsafe situation (*configuration*).

The security policy *enforcement* and *configuration* rule templates used in a profile are specified following an Event-Condition-Action (ECA) structure. Events represent activities in the IoT system that already took place or are about to take place but have not yet started, which are named respectively *actual* or *tentative* events. The distinction between actual and tentative events is necessary to enable the specification of detective and preventive policy rule templates. Detective rule templates are only able to react to actual events and execute additional compensation actions while preventive rule templates may allow, deny, modify, or delay the execution of tentative activities. Examples of reactive enforcements are simply the execution of additional activities in the IoT device or systems, for example, notifications to the users or compensation activities.

**Fig. 1:**
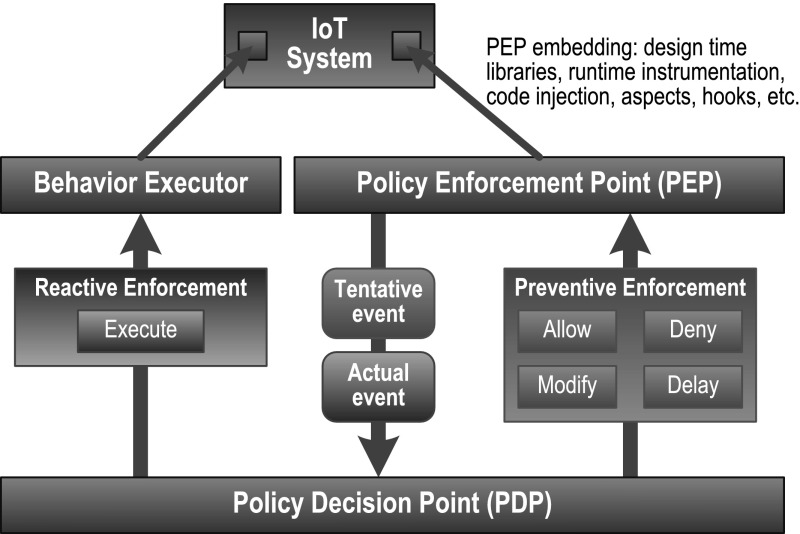
Enforcement Architecture

Figure 1 shows the security enforcement architecture that evaluates and enforces the ECA rules defined using the SecKit. The Policy Decision Point (PDP) receives event notifications from the Policy Enforcement Point (PEP) and evaluates the security policies. In response to the signaled events, the PDP replies to the PEP with a preventive enforcement message for tentative events or a reactive enforcement message to the Behavior Executor for actual events. PEPs are embedded in the IoT system using standard techniques such as custom libraries or runtime instrumentation. The PDP may also decide to deploy additional security policy rules in response to changes in the context or system events using dynamic policy configuration templates.

The PDP and PEP components of the toolkit are deployed and activated in the IoT devices either as part of the IoT device platform design, as part of the IoT application design, or as an add-on embedded transparently in the application or platform at runtime. Additional details on the deployment aspects of the toolkit are provided in “Deployment Aspects” section.

The ECA rule policy language supports the specification of templates with complex conditions including context situation events, propositional, temporal, cardinality, role assignments, and trust assessment operators. Rule templates are parameterized with variables and may be recursively nested using a flexible configuration rule that instantiates and disposes rule templates according to particular conditions. For example, a configuration rule may be specified to instantiate a set of enforcement templates when a particular context situation starts (e.g., moving from the house to a public environment) and to dispose the instantiated templates when the situation ends (e.g., exiting from the public environment). The change of the context can be simply triggered by a recording of location (i.e., provided by a GPS receiver or communication transmitter in the smartphone) or by other events like the authentication of the badge when an employee enters the office. This capability is important to support changes in the context and address challenge (11). A change in the context could also change the levels of access to the IoT services and devices as shown in “User Interface and Customization of Policies” section. The change of the levels of access is embedded in the policy template and the user has the power to accept, modify or reject it before the policies are deployed and activated in the IoT devices used by him or her. The toolkit can be used to address challenges (2) and (3) in Table 1, through policies, which automate or support the decision process in the interaction of the user with the IoT. In fact, as described in Kounelis et al. (2014), the toolkit can be used to define threat scenarios that provide an indication of the level of risk and required trust in a specific IoT operation including the possible negative consequences. The risk assessment model uses policy profiles as reference countermeasures that could be adopted to mitigate specific threat scenarios. For example, the toolkit can block a request from a malicious entity if it impacts the safety of a user as in the case of Smart connected cars (e.g., somebody hacking the car and interfering with the brake system).

Nested enforcement rule templates also specify a conflict resolution algorithm that should be used when two or more policy rules with conflicting results are triggered, such as first applicable in their specified order. This is important to address challenge (4) about the influence of Psychological bias. While policies created by users could have embedded personal bias, the conflicts with more generic rules (e.g., privacy regulations) will be detected and can be automatically resolved by the toolkit using the selected conflict resolution algorithm.

An important capability, which can be provided by the toolkit is to separate the On-Line and Off-line identity because the policy can be designed in such a way as to expose only specific information of the user in the on-line word, which can be separated by its real off-line identity. In other words, policies can be used to implement anonymization through pseudonyms or obfuscation of personal data (e.g., location data) to address the challenge (8) in Table 1. In other worlds, the toolkit can support the concept of “privacy by design”.

Additional details on the structure and capabilities of the Model-based Security Toolkit are presented in (Neisse et al. 2015). The SecKit framework has been implemented and already validated by some of the authors in (Neisse et al. 2014b) for feasibility and performance aspects in various IoT devices. It is available as open source software for download at https://github.com/r-neisse/Release.

### User Interface and Customization of Policies

While the SecKit can be a powerful tool to express policies, its value to support the Ethical Design concept is limited if there are no interfaces to generate, customize or modify profiles and templates in an easy and effective way. Users can select, customize parameters, modify, and define their own profiles according to their expertise. A pre-defined set of Profiles can be pre-generated for specific users (e.g., sportsman, elderly man), context situations (e.g., at work, at home), and service types (e.g., banking, online, social networking). For example, a policy template could be specified to regulate the authorization and data retention policy of a data item, and an activation profile could be specified to instantiate this template to the home when an online social network tries to access the user location. The customization of the profiles and the related policies can have limits imposed by regulations: policies can embed regulations defined by regulatory bodies, which can be enforced at all times (e.g., do not drive at an excessive speed) or in specific contexts (e.g., an emergency crisis) and specific roles of the user (e.g., a law enforcer). These policies may not be modifiable by the user as they could be defined to address societal needs. For example, in the case of an emergency crisis, law enforcers’ vehicles should have priority in traffic and vehicle drivers should not change this pre-defined policy. In other words, the toolkit can support the implementation of regulations and act on them especially in case of specific contexts (e.g., emergency crisis). The toolkit can also address inevitable changes in the regulatory frameworks in already deployed IoT systems and devices based on the toolkit because the software or hardware of the IoT devices does not need to be modified. When a new regulation must be enforced, new policies can be created and distributed to the IoT devices. This is an important element to address challenge (10).

As described previously for the challenge (9) Digital Divide in Table 1, not all the users of the IoT have the same technical proficiency. While experienced users could modify and define their own policy templates and instantiation profiles, other users could simply select and use profiles and templates proposed by certified companies or organized institutions (e.g., data protection organizations). More details on the different stakeholders involved in the deployment of the toolkit and the policies are provided in “Deployment aspects” section.

**Fig. 2:**
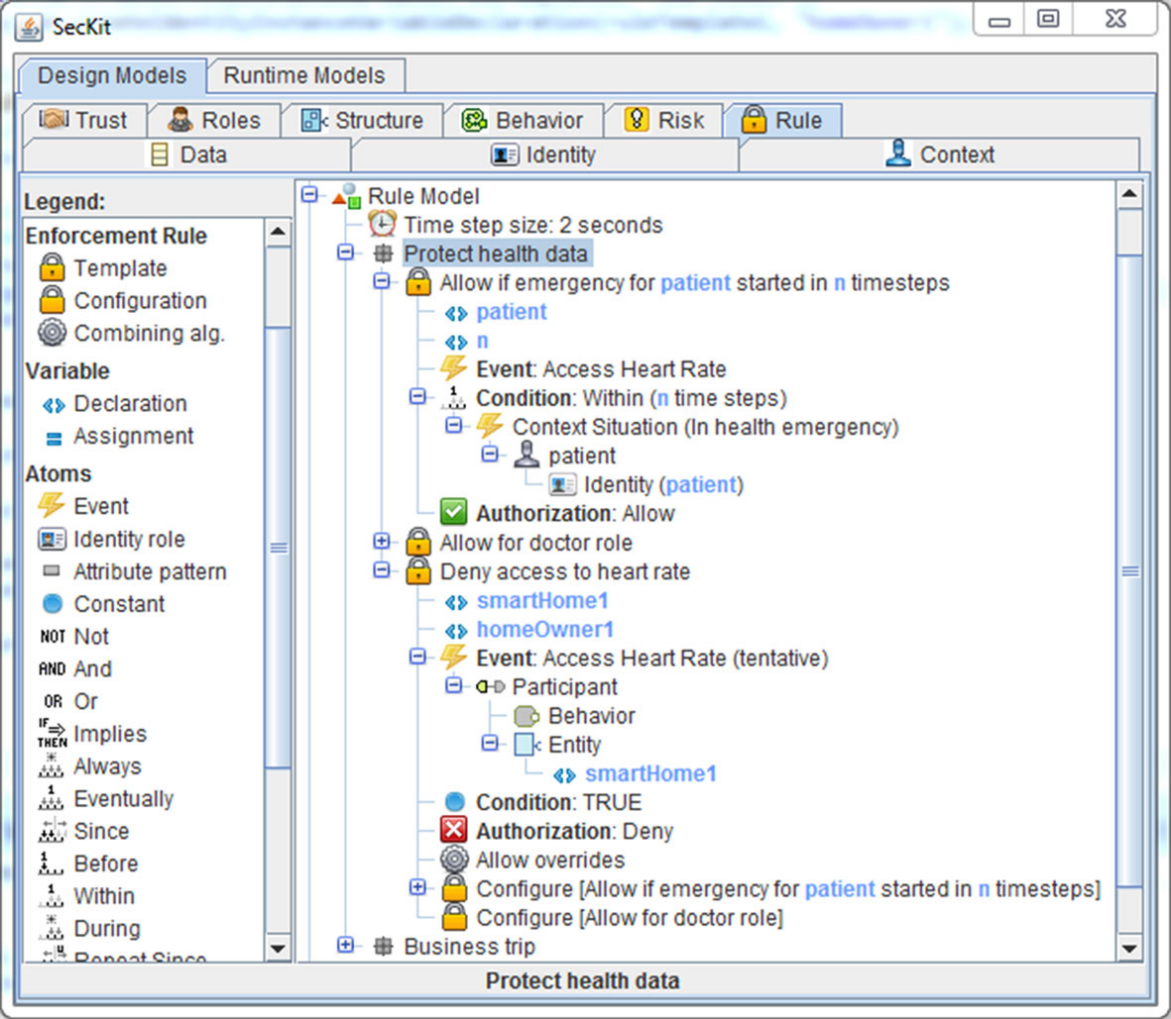
SecKit expert GUI for security policy profile authoring

Figure 2 presents the Graphical User Interface (GUI) available in the SecKit for the authoring of profiles by security policy experts. The profile *Protect* health data specifies three policy templates and illustrates the use of variables, nesting of enforcement template configurations, and combining algorithms. The GUI shows the case where an emergency situation generates a change in context where health information (e.g., heart rate) is made available when it is usually restricted only to authenticated and authorized doctors. Obviously the profile *Protect* has to be approved by the patient in first place. The first enforcement template allows access to the heart rate of a patient in case of a health emergency and the second enforcement template allows the access to the heart rate for doctors. These two enforcement templates are used in the third enforcement template, which denies access to the heart rate and specifies the *Allow overrides* combining algorithm, which will allow access if at least one of the nested rules evaluates to Allow. This example profile implements a whitelisting approach by blocking by default any access to the heart rate and allowing only doctors or in cases of emergency. For simplicities sake we do not show the details about the second enforcement template and configuration rules for instantiation of profiles and assignment of variables. Through the GUI, experienced users can modify the policy templates, while non-experienced users can use the services of certified bodies (see deployment aspects in “Deployment aspects” section).

In order to be meaningful, the policy configuration and enforcement rule templates presented to the user as part of a profile should be specified at a high level of abstraction, without including too many details about the underlying IoT technology and protocols. Ideally, profiles should be completely technology agnostic, and automated approaches to refine these abstract rule templates should be used. In a previous publication (Neisse and Doerr 2013) some of the co-authors propose a set of automated refinement rules that can be used to refine technology-agnostic user policies to enforceable technology-specific policies. These sets of refinement rules consider an abstract reference system and the refinement of the system design for technology specific solutions. In the solution proposed in this paper the IoT device manufacturers are required to provide details about their solutions in order to enable the automated refinement of technology-agnostic enforcement templates. The final goal is a semi-open IoT solution design that allows the enforcement of security policies to control the usage of sensitive user data or the IoT services as requested by the Ethical Design concept. The SecKit runtime enforcement elements that evaluate and enforce policies can be implemented and deployed in the IoT devices in a distributed manner.

### Deployment Aspects

**Fig. 3:**
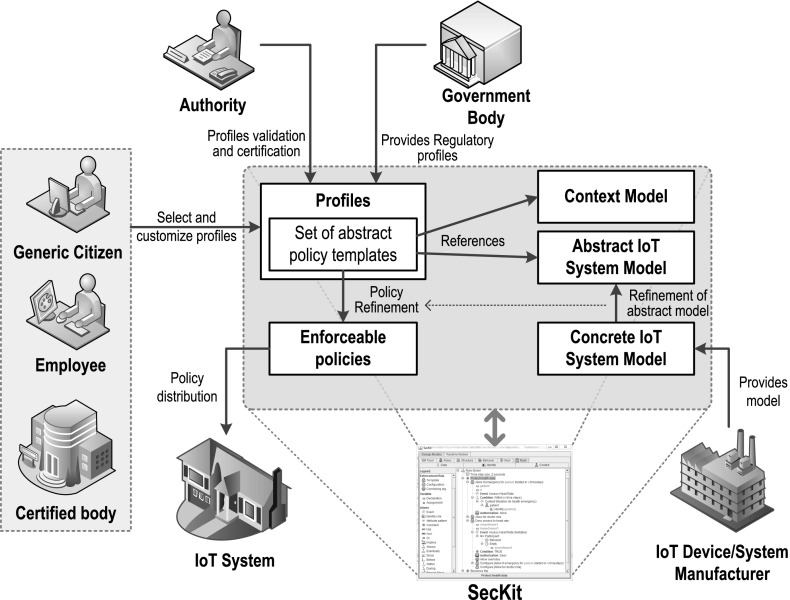
Pictorial description of the policy framework to support ethical design

In this section, we briefly describe the organization and deployment aspects of SecKit, which must be taken in consideration to foster the concept of Ethical Design. The overall schema for the generation, certification, deployment and customization of the policies is presented in Fig. 3 with the main stakeholders involved. The figure explicitly illustrates the role of the IoT device/system manufacturers in providing models of their systems that are used to support the refinement of abstract security policies into enforceable policies that can be distributed and deployed in concrete IoT system implementations.

The deployment of the SecKit can be based on different scenarios: (a) the SecKit can be embedded in the design of the IoT device, (b) in the design of the IoT application or (c) can be installed and activated for specific calls and data flows by intercepting the Application Programming Interface (API) calls or the data flows at runtime, before they are transmitted to/from the IoT and the network and cloud. The consequence of adopting one of the different deployment scenarios is the higher or lower degree of control provided to the toolkit and the user. The adoption of the scenarios depends on approach defined by the public and private decisions makers as discussed in “Challenges and processes for Ethical Design in the IoT” section. If the government and an IoT industrial forum decide that the toolkit (or an equivalent solution) must be part of the standard on which the design of the IoT device is based, scenarios (a) and (b) could be more likely. If no agreement is reached, scenario (c) can be adopted. Because the PEP is simply a runtime enforcer component, while the PDP evaluates the policy logic, we recommend that the PEPs are installed and activated in the IoT devices with lower computing processing power (e.g., an IoT sensor), while the PDP can be in the IoT device with higher computing processing power (e.g., the computing platform in a Smart Car or Connected Vehicle). The evaluation of the performance of the SecKit in the IoT devices and systems has been validated successfully as described in Neisse et al. (2014a, b).

Even if the SecKit itself is already available for download and use, organizations must be setup for the definition of policies and profiles and their distribution. Figure 3 provides a description of the main participating entities. As mentioned previously, policies and profile templates must be defined for different categories and users. These templates could be created by either public or private organizations (e.g., Government or industrial funded body), which must be certified to this purpose. Because profiles must embed regulatory frameworks, a translation of the regulatory framework must be executed and implemented in the policy templates for different types of context. Then a secure distribution channel of the policy templates must be set-up to ensure that they are correctly installed in the IoT devices without being tampered. The distribution and installation requires the involvement of the IoT Device or system manufacturers. The activation of the templates can be executed in the enrollment and registration phase of the IoT device (e.g., at the point of sale). Even if the SecKit is technology agnostic, the successful utilization of the template requires the definition of the concrete IoT system model linked to SecKit deployment. For example, the system model of a Smart Car will be different from the system model of a remote health monitoring system. The IoT system model is then formally described in the SecKit policy template. For example, we could have a SecKit policy template for the generic driver in a consumer Smart Car, where privacy risks (e.g., tracking of the car) or safety considerations could be mitigated through the application of the policy template.

### Analysis and Comparison with Other Frameworks

As recently pointed by a comprehensive survey on security, privacy, and trust in the IoT in (Sicari et al. 2105), the SecKit is the only IoT-specific solution able to guarantee the enforcement of security and privacy policies. Other policy-based solutions such as XACML (Rissanen 2010) could also be adopted; however, they only support authorization policies and have less expressive power supporting only propositional operators in contrast to the SecKit policy rule language that supports temporal and cardinality operators in addition to events, context, trust, and role operators. Using the XACML policy language a simple policy allowing access to the IoT device information only once per hour cannot be expressed. The SecKit policy language also has more expressiveness considering the enforcement actions, which not only include the possibility to allow or deny an activity but also it can express modifications and delays, for example, to anonymize information. Policy rules may also be nested and instantiated dynamically allowing modular specification and reuse of enforcement strategies.

The SecKit policy language is able to support most of the functions used to mitigate privacy risks: privacy, access control and data minimization. Access Control is supported by SecKit by defining policies, which regulate the access to data or services for specific types of users and roles. As described in “Policy-Based Approach for Ethical Design” section, it is also possible to implement access control depending on the context where the user operates. Data Minimization is supported by SecKit by defining policies, which minimize the data transmitted to other parties or to remote systems. For example, the profile chosen by the user could filter out the data, which identifies the person, and transmit only the sensor data. Through similar means, the SecKit could support data anonymization to mitigate privacy risks. For example, the identity of the user could be replaced by a pseudonym by the SecKit before the data is transmitted. A complete analysis of other IoT-specific and general purpose policy-based frameworks is already presented by some of the co-authors in Neisse et al. (2015).

A potential issue is that the framework can become very complex to design and maintain with the generation of many different profiles and different versions. On the other side, the complexity of the deployment of the framework is directly related to the complexity of the environment where the user operates. While such complexity cannot be avoided, the framework aims the help the user to address such complexity by providing an engineering tool (i.e., the SecKit itself), which automates the choices for the user. As described in “Policy-Based Approach for Ethical Design” and “Conclusions and Future Developments” sections, the toolkit can hide the complexity in using the IoT data and services in the field by automating the decisions of the users, which were already pre-selected and defined in the profiles chosen by the user. To summarize, the complexity of interacting with the IoT is embedded by the proposed framework instead of being a direct burden on the user. This concept is very important for the users, who are not proficient in the use of the IoT (i.e., the digital divide challenge described before).

## Conclusions and Future Developments

In this paper, we introduced the concept of *Ethical Design* with the aim of empowering the user in the interaction with the IoT. We have identified the main challenges in the evolution of Internet towards the IoT in order to establish interactions with the users that are respectful of their needs and privacy rights—namely to empower users. Then, we have described a potential implementation of the Ethical Design concept through a policy-based framework called SecKit, which has been already developed and whose source code is publicly available. We have shown how the framework can address all the identified challenges. We have also described the deployment aspects of the framework for a successful adoption of the Ethical design concept by the community. Such deployment will require the set-up of public and privacy entities for the definition of the policy templates, which can be customized by the users and certification processes and centers. The SecKit policy-based framework presented in this paper can give more control to the user and it can automate some of the complex decision processes in the interaction of the user with the IoT, but a complementary set of regulatory measures and best practices could make the application of SecKit more effective. For example, an increased awareness of the privacy risks in user communities is a non-technical recommendation, which could foster the adoption of the SecKit or similar frameworks. The management of data in large data centers is another area where new regulatory frameworks should be put in place or they should revised as the SecKit is more focused on the control of data coming from the IoT devices. There is currently an intense discussion among regulatory bodies on how to define adequate best practices or regulation for data management in large data centers especially when the data centers are cross-border. Finally, we note that the SecKit supports the user on his/her interaction with the IoT, but it does not address the infringement of citizen rights by malicious parties. In this case, existing regulatory and law enforcement processes must be applied or be defined.

Future developments will investigate the main regulatory and standardization processes to support the *Ethical Design* concept. In addition, we will research and identify security solutions, such as the application of cryptography solutions (e.g., PKI), to complement the policy-based framework for the secure distribution of the policy templates.
